# On the sensitivity of TG‐119 and IROC credentialing to TPS commissioning errors

**DOI:** 10.1120/jacmp.v17i1.5452

**Published:** 2016-01-08

**Authors:** Drew McVicker, Fang‐Fang Yin, Justus D. Adamson

**Affiliations:** ^1^ Medical Physics Graduate Program Duke University Medical Center Durham NC; ^2^ Department of Radiation Oncology Duke University Medical Center Durham NC USA

**Keywords:** TPS commissioning, IROC, RPC, IMRT, AAA

## Abstract

We investigate the sensitivity of IMRT commissioning using the TG‐119 C‐shape phantom and credentialing with the IROC head and neck phantom to treatment planning system commissioning errors. We introduced errors into the various aspects of the commissioning process for a 6X photon energy modeled using the analytical anisotropic algorithm within a commercial treatment planning system. Errors were implemented into the various components of the dose calculation algorithm including primary photons, secondary photons, electron contamination, and MLC parameters. For each error we evaluated the probability that it could be committed unknowingly during the dose algorithm commissioning stage, and the probability of it being identified during the verification stage. The clinical impact of each commissioning error was evaluated using representative IMRT plans including low and intermediate risk prostate, head and neck, mesothelioma, and scalp; the sensitivity of the TG‐119 and IROC phantoms was evaluated by comparing dosimetric changes to the dose planes where film measurements occur and change in point doses where dosimeter measurements occur. No commissioning errors were found to have both a low probability of detection and high clinical severity. When errors do occur, the IROC credentialing and TG 119 commissioning criteria are generally effective at detecting them; however, for the IROC phantom, OAR point‐dose measurements are the most sensitive despite being currently excluded from IROC analysis. Point‐dose measurements with an absolute dose constraint were the most effective at detecting errors, while film analysis using a gamma comparison and the IROC film distance to agreement criteria were less effective at detecting the specific commissioning errors implemented here.

PACS number: 87.55.Qr

## I. INTRODUCTION

In radiation therapy, proper commissioning of the dose calculation algorithm in the treatment planning system (TPS) is essential because any errors in this process impact all treatment plans prepared in the system. Proper quality assurance (QA) procedures must be in place in order to ensure this commissioning process. The AAPM publication by Task Group 53 is a guideline for this commissioning process, and provides the framework to allow physicists to design comprehensive and practical treatment planning QA programs for their clinics without prescribing specific tests.^(1)^ In addition, the report by Task Group 119 (TG‐119) furthered this initiative by producing quantitative confidence limits as baseline values for commissioning IMRT planning and delivery systems.^(2)^ Another means to verify the TPS and IMRT commissioning process is through credentialing processes offered by outside institutions. The most common interinstitutional credentialing is through the Imaging and Radiation Oncology Core (IROC, formerly the RPC) which provides phantoms equipped with point measurement dosimeters and film planes to verify the proper dose delivery based on provided constraints.^3^, ^4^

A number of studies have evaluated the clinical effect of various errors including those introduced during TPS commissioning.^5^, ^6^, ^7^, ^8^, ^9^, ^10^ For instance, Rangel et al.^(5)^ determined tolerances for beam modeling based on equivalent uniform dose (EUD) in clinical plans using multiple beam models in the Pinnacle TPS. A number of investigators have studied the clinical consequences of systematic and random MLC errors, including MLC calibration errors for 3D CRT, IMRT, and VMAT.^6^, ^7^, ^8^, ^9^ Other studies have also evaluated the sensitivity of 1D and 2D detectors to commissioning and delivery errors.^(10)^ Despite these studies, there is little published data regarding the sensitivity to commissioning errors of the IMRT commissioning procedures described in TG‐119 and credentialing via the IROC.

The purpose of this study was to investigate the ability of the TG‐119 guidelines for IMRT commissioning and IROC credentialing to detect errors in the commissioning process of IMRT within a commercial treatment planning system (TPS). We evaluated the sensitivity of a subset of the TG‐119 geometries and the IROC head and neck credentialing phantom to various commissioning errors introduced in a modern dose calculation algorithm (analytical anisotropic algorithm or AAA, version 8917) within a commercial TPS (Eclipse vs. 8.6, Varian Medical Systems, Palo Alto, CA).

## II. MATERIALS AND METHODS

### A. Overview

**Figure 1:**
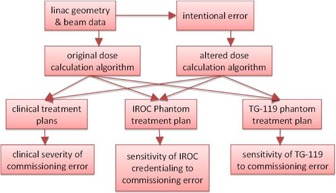
Workflow to determine the clinical severity of commissioning errors along with the sensitivity of IROC credentialing and IMRT commissioning from TG‐119.

As illustrated in Fig. 1, to measure each QA technique's sensitivity and ability to detect errors, first various errors were implemented in the Eclipse analytic anisotropic algorithm during the commissioning process for a Varian 2100 series linear accelerator (21EX, Varian Medical Systems). Each error was implemented individually to isolate its effect. Once the algorithm was commissioned with an intentional error, the dose for select clinical plans was recalculated using the altered algorithm and compared to the original plans to measure the clinical impact that the commissioning error would have. To measure the sensitivity of the IMRT commissioning process and IROC credentialing, the same was done with plans designed on the TG‐119 C‐shape^(2)^ and IROC head and neck IMRT phantoms.^3^, ^4^ The newly calculated plans were then compared to the original plans to determine the dosimetric effect of each error.

### B. Treatment planning system

The AAA commissioning process within the Eclipse TPS has three steps: 1) data entry, 2) calculation of the beam model, and 3) verification of the beam model.^11^, ^12^, ^13^ Table 1 shows each of the data elements required for the commission procedure and where they fall in terms of dose contribution. The data required to calculate the beam model come from a combination of data entered manually by the user (e.g., beam geometry, output factors, depth dose curves, beam profiles, flattening filter material) and data retrieved from a machine data library (mean radial energy, initial photon energy spectrum). Primary parameters are those used to model the primary source of photons, which consists of Bremstrahlung X‐rays produced in the target that do not interact in the head of the treatment unit. Secondary parameters model dose from photons scattered from the flattening filter and collimators, which is modeled as a virtual plane source located at the bottom of the flattening filter. Electron contamination parameters model dose from electrons generated in the treatment unit head and in air, and is modeled at the target plane using two Gaussian curves (which determine lateral spread of electrons and field size dependence) and one dose deposition curve, which is derived empirically by the beam modeling algorithm as the difference between the largest field size depth dose curves measured with and calculated without the electron contamination. While the primary photon, secondary photon, and electron contamination are defined, modeled, and verified within the AAA commissioning procedure, the MLC parameters are not included in this process and are, instead, defined separately from the beam model.

During the first step of the commissioning process, machine parameters are input and, using these parameters, the TPS accesses a machine library that includes information about the specific machine that is being commissioned. Step 2 (calculation of the beam model) occurs once the required data have been entered. During this step, the beam model parameters, such as the initial beam spectra and second source parameters, are fit to match the input beam profiles and depth curves. After the AAA beam modeling algorithm is prepared, the TPS allows the user to evaluate the agreement between the calculated and measured curves. This comparison is done for all input beam profiles and depth‐dose curves.

**Table 1:** Treatment planning system commissioning parameters for AAA.

*AAA Dose Calculation Components*	*TPS Parameters*	*Errors Implemented*
Primary Photons	Output Factors	Incorrect SAD
	Depth Dose Curves	Flattening Filter Material
	Beam Profiles	TMR input as PDD
	Spectrum Parameters	Smoothed Dose Profiles
Secondary Photons	Mean Radial Energy	
	Size of Second Source	
	Relative Intensity	
	Energy of Second Source	
Electron Contamination	Electron Contamination $\sigma_{0}$	Change in $\sigma_{1}$
	Electron Contamination $\sigma_{1}$	
	Relative Fraction	
MLC Parameters	Dosimetric Leaf Gap	MLC DLG Error
	Transmission Factor	Transmission Factor Error

### C. Implementation of errors

We introduced commissioning errors into all four AAA dose calculation components listed in Table 1: primary photons, secondary photons, electron contamination, and MLC parameters. Below we discuss the commissioning errors introduced in detail.

For the primary and secondary photons, errors included entering the incorrect source‐to‐axis distance (SAD), incorrect flattening filter material, incorrect input of tissue‐maximum ratio (TMR) instead of percent depth‐dose (PDD) curves, and smoothing the input dose profiles (using an arithmetic mean smoothing method with a sliding window size of 6 mm) to simulate measuring the beam data using an inappropriately large Farmer chamber. The AAA beam modeling algorithm allows the user to select the flattening filter material, which in turn determines the initial beam spectra for the beam model calculation. We changed this material from the default (copper) to each of the other possible flattening filter materials (tungsten, lead, and iron). To select an incorrect material, the user must manually skip a step in the commissioning process that retrieves parameters from the machine library.

For the electron contamination component, electron contamination fluence is simulated within the dose calculation algorithm using a convolution of aperture shape and a 2D sum‐of‐Gaussians kernel.^11^, ^12^, ^13^ The user‐defined parameters include two different sigma values that model the lateral distribution for each Gaussian curve and a relative fraction specifying the weight of each Gaussian. During the commissioning procedure, initial values for these are obtained automatically from the machine library; the initial values are 69.99 mm and 99.99 mm for $\sigma_{0}$ and $\sigma_{1}$, respectively, and a relative fraction of 0.4384. To alter these values, the user must skip a step of the configuration process so that the values are not reset. For this project, we started out by implementing a very large error (70 mm) in $\sigma_{0}$ to see what effect it had on clinical plans. In this case, the effect turned out to be fairly subtle; hence, smaller magnitude changes were not applied since their effect is expected to be negligible.

For the MLC parameters, both the dosimetric leaf gap (DLG) and leaf transmission were modified. The Eclipse TPS models the complex interactions of a beam incident upon the MLC leaves such as lateral disequilibrium,^(14)^ tongue‐and‐groove effect, and rounded leaf‐end effect by assuming a gap (DLG) between the ends of leaf pairs. The magnitude is set by the user; the nominal value of the DLG can be measured by comparison of line profiles of delivered and calculated dose.^(15)^ This value is then modified iteratively so as to obtain an accurate match between calculated and measured dose for representative IMRT treatment plans. In our case, the final DLG obtained after iterative comparison was 1.8 mm; we then implemented errors ranging from ± 0.1 to ± 1 mm from the nominal value. The MLC transmission factor models leakage dose through the MLC leaves. For our linear accelerator model, the original transmission value was 0.017, representing that 1.7% of the dose delivered is transmitted to areas blocked by the MLC leaves. We increased the transmission factor by 0.5% and 0.9% for a transmission factor of 0.022 and 0.026, respectively.

For many of the commissioning errors introduced, the error magnitudes that were chosen are arbitrary; for DLG the low and high magnitudes introduced were roughly 5% and 55% of the nominal value. For leaf transmission, we introduced roughly a 0.5% and 1% change in leaf transmission. It should be noted that the choice of magnitude is not so important because magnitudes are used to evaluate both the effect on clinical treatment plans and the discrepancy introduced in the IROC and TG‐119 phantoms. More important is the relative difference between sensitivity to the error in clinical plans and the IROC/TG‐119 phantoms. For this reason we chose a “low” and “high” error magnitude, and the effect at other magnitudes can then be inferred.

### D. Clinical impact

To determine the clinical impact of the commissioning errors, we determined their effect on clinical dose metrics for five representative clinical treatment plans. The plans used included a low risk prostate, an intermediate risk prostate, a head and neck (right sided), a mesothelioma (right sided), and a scalp case. The treatment plans were chosen to be a representative sample of the types of IMRT plans to be created by the planning system. The mesothelioma plan was included because of its ability to test dose calculation in the presence of tissue heterogeneities. The scalp case was included specifically for investigating electron contamination parameters, since the other plans were largely unaffected by electron contamination. The head and neck case and the scalp plan were the only plans used to analyze changes in electron contamination metrics as they were the only cases with critical structures within the electron range distance from the surface. Each of these plans has different source‐to‐surface distances (SSD), target volume sizes, and organs at risk near the treatment volume, but all plans used a 6X photon beam for treatment. Clinical dose metrics evaluated included $D_{99\%}$ and $D_{1\%}$ of the PTVs, as well as mean or max dose of nearby critical organs depending on whether or not those organs were serial or parallel. The mesothelioma case was used primarily to determine the effects of commissioning errors in low‐dose regions, with relevant dose statistics being the volume of the nontreated lung to receive dose over 5 Gy, 13 Gy, and 30 Gy, as well as the volume of the heart to receive over 38 Gy or 42 Gy. The prostate plans required the deepest penetration of any of the plans considered, and were used in determining the impact of the implemented errors at depth. The original head and neck, mesothelioma, and scalp treatment plans are illustrated in 2, 4.

**Figure 2:**
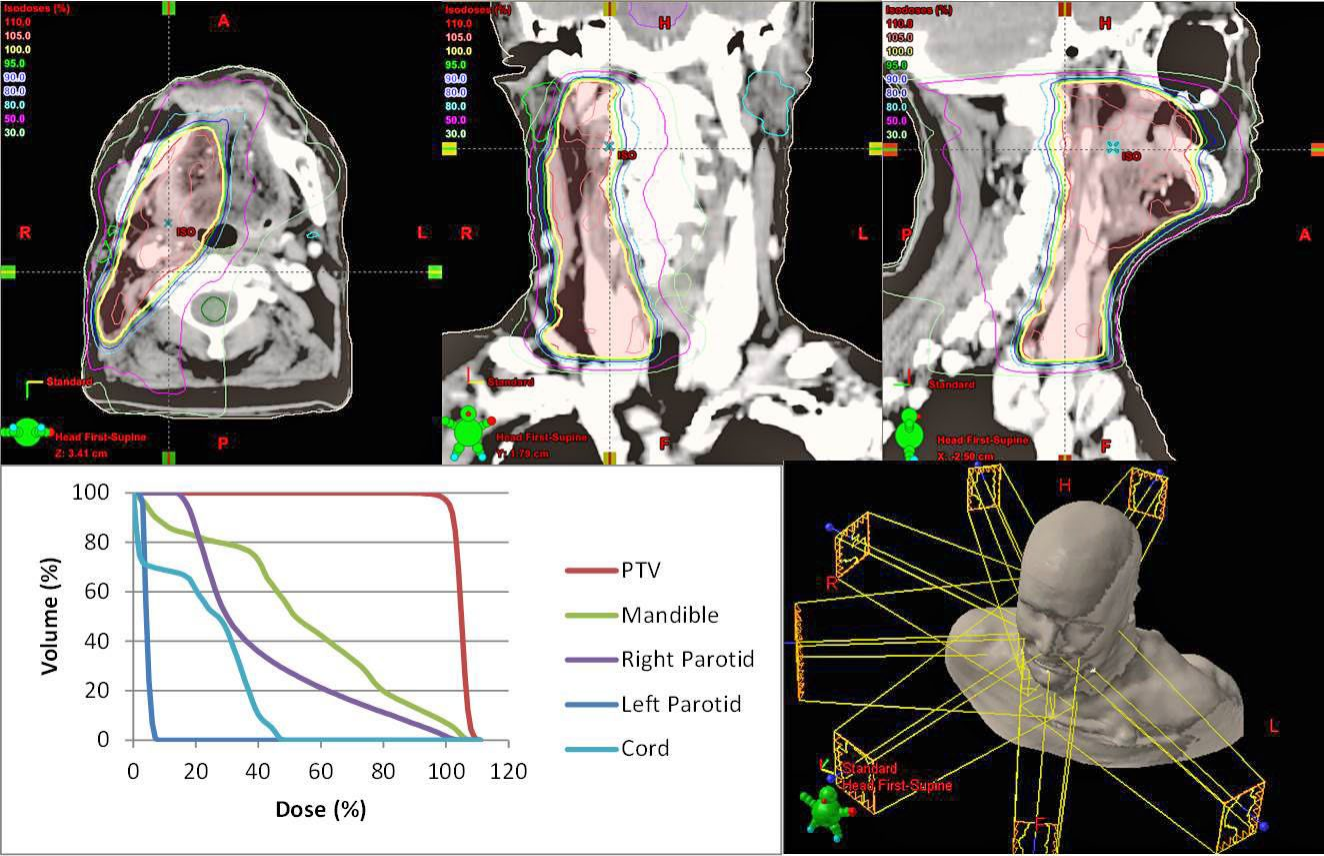
3D rendering, axial, coronal, and sagittal dose distribution and DVH of head and neck IMRT plan.

**Figure 3:**
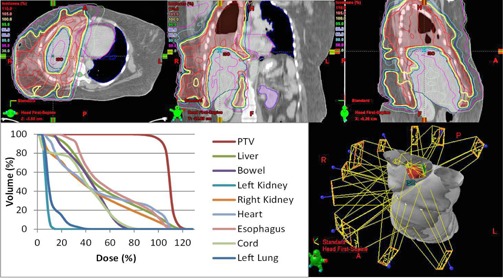
3D rendering, axial, coronal, and sagittal dose distribution and DVH of mesothelioma IMRT plan.

**Figure 4:**
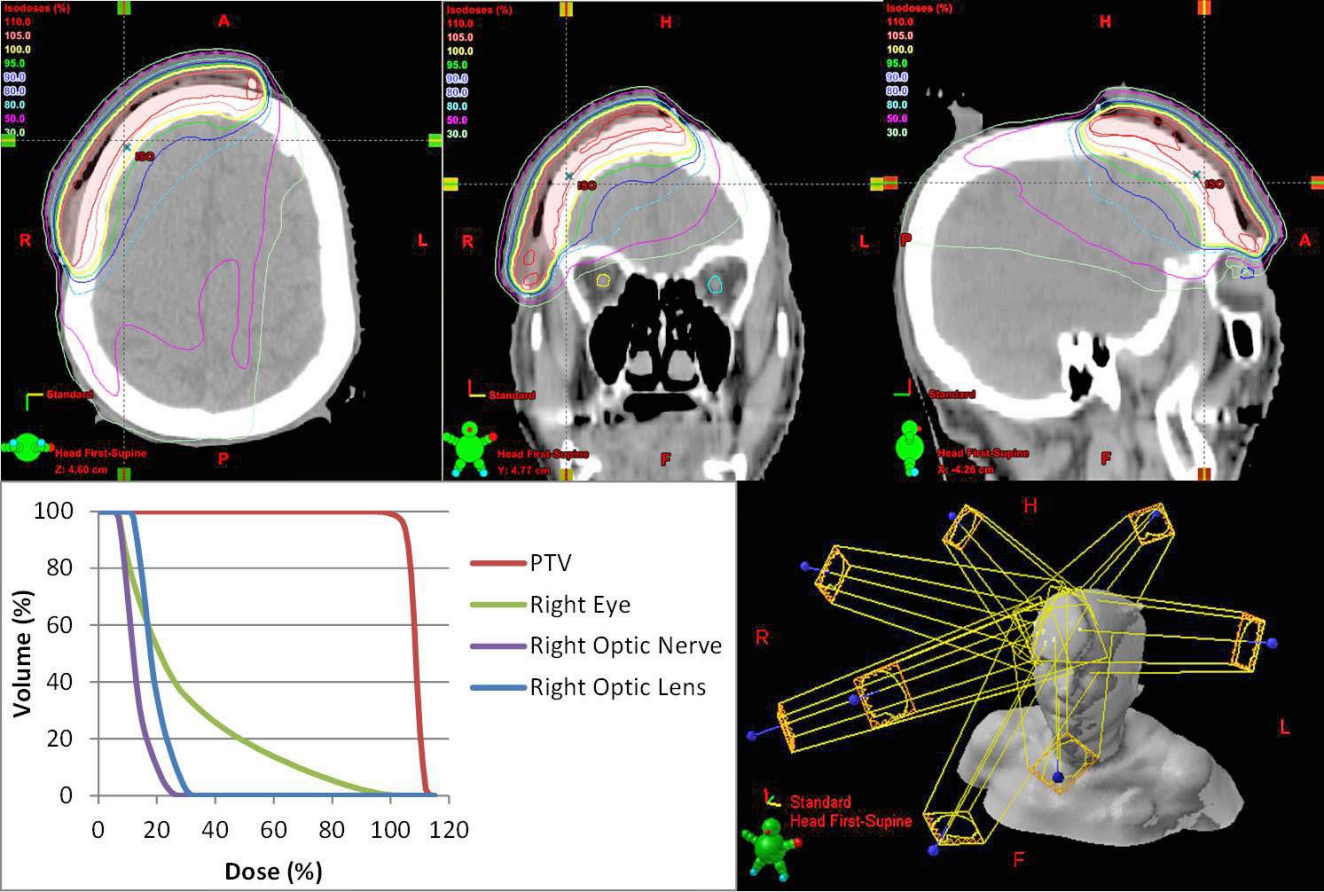
3D rendering, axial, and sagittal dose distribution and DVH of scalp IMRT plan.

### E. Sensitivity to errors of TG‐119 C‐shape phantom

**Figure 5:**
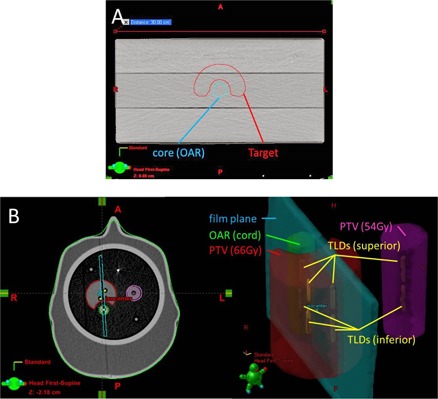
Axial view C‐shape structures (a) from TG‐119 suite, superimposed on a rectangular phantom. IROC phantom geometry (b) for targets, critical structure, film plane, and TLD locations.

The sensitivity of the TG‐119 IMRT commissioning process using the C‐shape geometry^(2)^ was evaluated by recalculating the dose distribution of a previously optimized plan using the altered AAA beam model. For the C‐shape phantom, the optimization constraint required the central core structure to received <50% of the prescribed dose. The TG‐119 C‐shape phantom is assessed using three planar film measurements and evaluated using a two‐dimensional gamma analysis with the criteria of 3% dose/3 mm distance to agreement. An axial view of the C‐shape geometry superimposed on a rectangular phantom is shown in Fig. 5(a); further details are available from the AAPM Task Group report and online materials.^(2)^

### F. Sensitivity to errors of IROC credentialing

During the IROC credentialing process with the IMRT head and neck phantom, the IROC analyzes the institution using point doses currently measured by eight thermoluminescent dosimeters (TLDs). Four are located in the primary PTV, two in a secondary PTV, and two in the OAR (one superior and one inferior). To pass the IROC credentialing, the point‐dose measurements within the PTVs must be within 7% of the expected value. While all TLDs are measured, the IROC passing criteria only applies to the TLDs within the PTVs. A sagittal film is also measured and evaluated by the IROC, using a 4 mm distance‐to‐agreement metric for the midpoint dose between the PTV and OAR. The ability of IROC credentialing process to detect commissioning errors was tested by comparing the dose distribution calculated to the IROC head and neck phantom before and after implementing a given commissioning error. The IROC phantom geometry is shown in Fig. 5(b). Further details for the IROC phantom geometry are given elsewhere.^3^, ^4^

## III. RESULTS

### A. Overview

**Figure 6:**
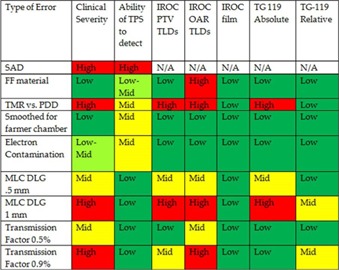
Summary of clinical severity and probability of detection for errors implemented in Eclipse AAA.

Figure 6 summarizes the clinical severity of the various commissioning errors and probability of the errors being detected by the TPS during beam model commissioning process. It also includes the probability of their being detected during the TG‐119 IMRT commissioning process with the C‐shape geometry and during credentialing through the IROC. Clinical severity and detection probability were classified as low, medium, or high using the following criteria. Clinical severity and TLD detection was graded as “Low” if the effect was <2%, “Mid” if the effect was between 2% and 7%, and “High” if the effect was greater than 7%, which would fail the IROC credentialing criteria for the TLD measurements. The ability of the TPS to detect errors was graded as “Low” if there was no indication from the TPS that an error occurred, “Mid” if the error could be detected visually during the verification stage but no warning was given, and “High” when the system gave a visual warning and did not allow the user to continue. In the special cases of electron contamination errors, a ranking of “Low‐Mid” was assigned because it only had a nonnegligible (2.1%) effect on one of the five clinical plans analyzed (the scalp plan). A “Low‐Mid” ranking was also assigned to the ability of TPS to detect flattening filter material errors as a step of the commissioning process in which parameters are retrieved from the machine library must be skipped for the error to be implemented.

### B. Clinical impact

#### B.1 Incorrect SAD

The TPS invoked an internal error and prohibited the user from proceeding after an incorrect SAD was entered. Changes to the source‐to‐surface distance are expected to have a large dosimetric effect if they were to be implemented.

#### B.2 Flattening filter material

The TPS automatically reset the flattening filter material to the proper value when retrieving parameters from the machine library. If the user manually overrode the retrieval from the machine parameter library, the AAA beam modeling algorithm still compensated for the incorrect flattening filter setting by changing other parameters — specifically size, relative intensity, and mean energy of the second source. As a result, the beam model matched the input profiles, with the exception of the penumbra region where up to a 5% difference occurred. The clinical impact of changing the flattening filter in the TPS was minor in all of the plans considered, resulting in a mean change of within 0.3% in all of the clinical plans and a maximum change of 2.3% in the parotids of the head and neck plan, which is in a low‐dose region (<9% of the prescription dose).

#### B.3 TMR input as PDD

Clinically, input of TMR instead of PDD was the most severe of the errors implemented into the commissioning process. Importing TMRs instead of PDDs caused a mean difference in dosimetric indices of over 13% in prostate plans and differences of over 5% in all plans, with the least sensitive being the scalp case. This is unsurprising, as the area of interest in the scalp case is near the surface and the largest discrepancies between TMR and PDD occur at deeper depths.

#### B.4 Inappropriately large Farmer chamber

The smoothed dose profiles went relatively undetected by the TPS. Similar to the incorrect flattening filter material, differences could be detected during the verification stage when comparing the penumbra region of the dose profiles where there was up to a 10% difference. In general the clinical effect was minimal and within fractions of a percent, with the exception of the V_20Gy_ in the untreated left lung in the mesothelioma case which saw a change of more than 6% its original value. The effect of smoothing was minimized within the TPS because the calculated profiles matched the true profiles better than they matched the input (smoothed) profiles. While the AAA beam modeling algorithm attempts to match the calculation with the measured profiles, the differences in the penumbra region show that the TPS partially corrected the user error and decreased the effect that using an inappropriately large Farmer chamber would have on the dose calculation algorithm.

#### B.5 Electron contamination

Electron contamination was the most difficult error to implement into the system. The TPS normally retrieves the electron contamination parameters from the machine library, so to make any alterations to the parameters requires that the user prevent the TPS from searching in the machine library. After the user skips this step and makes a change to the parameters, the user must also skip the second step of the configuration process to prevent the values from being changed back to their original values. It therefore requires that multiple errors be made by the user, or alternatively that the values retrieved from the library be corrupted. Upon calculation of the percent depth‐dose curves and dose profiles, a difference of up to 20% is observable in the surface dose in the PDD for changes of 70 mm in $\sigma_{0}$. The only plan that saw any considerable clinically relevant dosimetric effect was the scalp case, with a maximum dose difference of over 2% in the mean dose to the right lens. Hence commissioning process for AAA in Eclipse is robust to electron contamination errors as they require multiple errors and input of values very far from the original values, and there is only a clinical effect of such errors for a PTV or OARs neat the skin surface.

#### B.6 Dosimetric leaf gap

**Figure 7:**
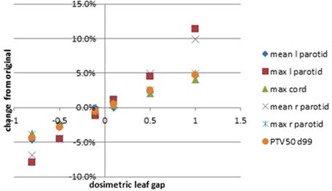
Display of change in dose from original head and neck plan due to changes in the MLC DLG.

The MLC DLG is entered separately from the AAA beam modeling algorithm, hence errors in the DLG were the least likely to be detected by the TPS. The input of an incorrect DLG was straightforward, and an improper DLG value was undetectable during the verification stage. The relationship between DLG error and relevant dosimetric indices for the head and neck plan is shown in Fig. 7. The relationship between the magnitude of change in the DLG value was proportional to the dosimetric effect induced by the error, with the slope of correspondence between the magnitude of error and dosimetric effect varies between organs. The most sensitive organs to changes in the DLG were the parotid glands, in which a 2% dosimetric effect occurred with a change in DLG of 0.2 mm and a 5% dosimetric effect with a change of 0.5 mm. Prostate plans, however, were less sensitive to changes made in DLG values, with all dosimetric indices remaining within 2% for 0.5 mm changes. This is true for the PTVs of all plans, with the only dosimetric indices outside of 2% being the lens and optic nerve in the scalp case.

#### B.7 MLC transmission factor

Similar to DLG, errors in the MLC transmission factor value are undetectable during the verification stage and the magnitude of the error is proportional to its dosimetric effect. The most sensitive plans to errors in transmission factor were the head and neck plan and scalp plan. In the head and neck plan, a 0.5% and 0.9% change in transmission factor resulted in a 4% and 7% change to the mean dose to the right parotid, respectively. In the scalp plan, a 0.5% and 0.9% change in transmission factor resulted in a 5% and 8% difference in the maximum dose to the right optic nerve, respectively. The prostate and lung plans were less sensitive, with these same changes in transmission factor resulting in changes of less than 2% in relevant dosimetric indices.

### C. TG‐119

Table 2 shows the percent of pixels with gamma index <1 comparing three orthogonal calculated dose planes due to the commissioning errors in the TG‐119 C‐shape phantom. Incorrect flattening filter, smoothed dose profiles (inappropriate large chamber), and changes to the electron contamination parameters did not cause any detectable difference in the dose planes of the TG‐119 C‐shape. Since the effect on clinical treatment plans was minimal for these errors, it is not surprising that they have little effect on the C‐shape geometry. As expected, the commissioning error with the largest effect on the C‐shape plan was the input of TMR curves instead of PDDs; in this case < 50% had a gamma index <1 when using a criteria of 3% absolute dose and 3 mm. Surprisingly, when the criteria were relative dose comparisons (both dose planes normalized to max values), the pass rates increased to 95% for all planes. The same was true for DLG errors analyzed with gamma index and relative dose criteria for which a 0.5 mm change in DLG went completely undetected with a 100% pass rate. This emphasizes the need for an absolute, rather than relative, dose comparison, as a relative comparison is insensitive to even drastic commissioning errors.

**Figure 8:**
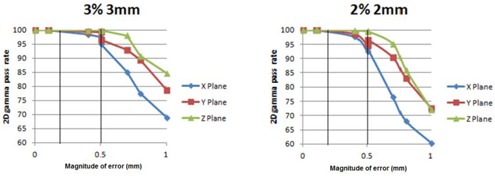
Percent of pixels in dose planes with gamma index <1 using 3%/3 mm and 2%/2 mm criteria as functions of magnitude of DLG commissioning error. Vertical lines represent the magnitude of DLG error that caused a 2% and 5% dosimetric error in select clinical treatment plans.

Figure 8 shows the percent of pixels in each dose plane which had a gamma index <1 as a function of the magnitude of DLG error. This figure shows the potential improved sensitivity when the gamma index criteria are changed from 3%/3 mm to 2%/2 mm. The vertical lines in Fig. 8 represent the DLG error which caused a change in relevant dosimetric indices for the clinical plans of 2% and 5%, respectively. It is clear from this figure that a DLG commissioning error that causes a 5% effect on clinical plans will still have a high pass rate for a film plane comparison of the TG‐119 C‐shape using gamma index with criteria of 3% absolute dose and 3 mm. The gamma index pass rates for this magnitude of DLG error are within the standard deviation of pass rates among treatment centers that submitted their results for the TG‐119 report.^(2)^

**Table 2:** Pass rates (mean ± standard deviation) for various gamma criteria for each error in TG‐119 film planes.

*Type of Error*	*3%/3 mm Absolute Gamma Analysis*	*2%/2 mm Absolute Gamma Analysis*	*3%/3 mm Relative Gamma Analysis*
Incorrect FF material	100%±0%	100%±0%	100%±0%
TMR vs. PDD	53.37%±6.10%	44.97%±4.19%	98.13%±2.34%
Smoothed for Farmer chamber	100%±0%	100%±0%	100%±0%
Electron Contamination	100%±0%	100%±0%	100%±0%
MLC DLG .5 mm	98.03%±1.96%	96.17%±1.96%	100%±0%
MLC DLG 1 mm	77.43%±7.97%	68.4%±6.84%	93.07%±8.22%
Transmission Factor 0.5% change	95.89%±3.55%	95.05%±4.05%	97.33%±2.29%
Transmission Factor 0.9% change	92.02%±5.16%	88.72%±6.49%	95.15%±3.92%

### D. IROC

**Figure 9:**
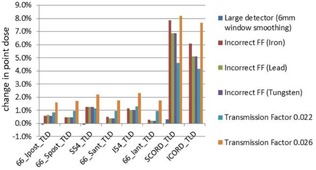
Percent change in TLD point dose in IROC phantom due to various commissioning errors.

**Figure 10:**
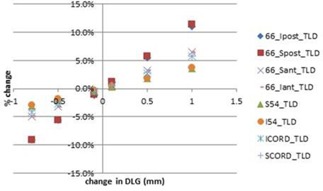
Sensitivity of TLD point does measurements to DLG errors in IROC IMRT phantom.

9, 10 show the sensitivity of IROC TLDs to various commissioning errors. While the PTV TLDs were effective in detecting clinically relevant errors such as the DLG errors (as shown in Fig. 9), Fig. 10 shows that the OAR TLDs are more sensitive to errors in flattening filter material and MLC transmission factor. IROC TLD measurements were effective in detecting TMR errors, as there was a mean difference of 10% between the original plan and the TMR plan.

**Figure 11:**
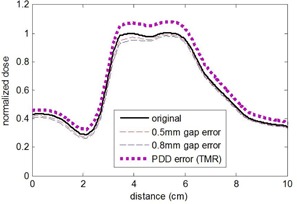
Dose profile from TPS across IROC film plane with various commissioning errors introduced. The IROC sagittal film analysis passed the 4 mm distance criteria for all commissioning errors implemented.

In addition to the TLDs, the IROC also includes the sagittal film plane analysis. All commissioning errors, including all DLG errors, flattening filter errors, and profile smoothing, and use of TMRs rather than PDDs, each fell well within the 4 mm criteria with no distance to agreement greater than 1 mm. The dose profile across the IROC film is shown for various commissioning errors in Fig. 11.

## IV. DISCUSSION

In our observation, the AAA was quite robust at preventing errors from being implemented. Many errors were prohibited by the TPS because it primarily relied on values stored in the machine library rather than user input (in the case of second source parameters), or detected input that was geometrically inconsistent (in the case of SAD changes). Beyond not allowing certain changes, the AAA commissioning process was able to correct some errors in the initial parameters because the beam modeling process fit the parameters to match the input beam profiles. For example, when an incorrect flattening filter material was selected, the final calculated beam profiles and depth‐dose curves still matched the measured data within 5% at all points throughout the curves. Also, some errors in the input profiles are negated because the commissioning process utilizes Monte Carlo‐based kernels from the machine library. For example, in the case of the overly smoothed dose profiles, the calculated profiles were more closely aligned to the actual beam profiles in the penumbra region than to the smoothed profiles.

The results provided here are specific to the AAA algorithm as implemented in the Eclipse TPS, and are applicable to other centers with the same TPS and algorithm. Analysis with other common TPSs would also be beneficial, especially when the commissioning process varies from that of AAA in Eclipse.

Uncertainty in remote TLD measurement has been found to be 1.5%.^(4)^ Using this statistic, the IROC has set an action criterion for remote output measurements at 5%, given the fact that there is less than 1% chance that the error of a single measurement is greater than 5%. For the IMRT head and neck phantom, the action criterion is increased to 7%. For comparison, we found that discrepancies detected in the IROC TLDs are roughly equivalent to the discrepancies occurring in clinical plans. Hence, the current 7% action criteria would likely translate into an equally large discrepancy in clinical plans. If only the PTV TLDs are analyzed (as is currently the case), then, for specific commissioning errors (transmission factor), the discrepancy in clinical treatment plans may be expected to be even larger than this action criterion.

TLD point dose measurements, along the critical structure meant to be avoided, are measured but not analyzed during the IROC credentialing process. Our results indicate that these TLDs were the most sensitive to detecting certain commissioning errors. The disadvantage of these OAR TLDs is that they can be overly sensitive to errors that clinically had minor effects. In order to detect the errors in electron contamination parameters or MLC transmission factor, dose measurements would need to be made in low‐dose regions and, in the case of electron contamination, near the surface.

TG‐119^(2)^ and the IROC^3^, ^4^ both utilize 1D and 2D measurements to evaluate agreement. Three‐dimensional dosimetry techniques that can sample an entire dose distribution with high spatial resolution have been applied to IMRT^16^, ^17^, ^18^, ^19^ and may be well suited for TPS commissioning and credentialing procedures for a number of reasons.

1. If shown to be more sensitive, the additional effort required to perform 3D dosimetry over less comprehensive measurements may be warranted due to the critical need for correct commissioning of the TPS and the potential severity of errors committed during this process.
2. Both the TPS commissioning and credentialing processes are carried out on an infrequent, rather than on a recurring, basis; hence the additional effort for 3D dosimetry would not affect the clinic workload of recurring quality assurance.
3. In the case of IROC credentialing, the phantom, film planes, and TLDs are already transferred to a central location; hence, the technology required to reconstruct and digitize the 3D dose cloud would not need to be disseminated on a widespread scale.

However the question remains as to how much benefit 3D dosimetry techniques would provide over current 1D and 2D measurements.

Recently, an effort has been made to failure mode and effects analysis (FMEA) to the field of radiation therapy as a means of reviewing the many components of the treatment process that could go wrong.^(20)^ FMEA is a primarily qualitative analysis of a complex system that is a systematic technique for failure analysis. It involves three steps:

1. The probability that a specific cause will result in a failure mode;
2. The severity of the effects of a failure mode should it go undetected, and
3. The probability that the failure mode resulting from the specific cause would go undetected.

FMEA is able to account for each possible cause of failure in a system and, if applied to IMRT, could serve as an effective means of identifying the cause of failures or errors, depending on their probability of occurrence and probability of detection at each step of the process.^(12)^ Our results have implications for this process: specifically, Fig. 6 may be useful when assessing severity and probability of various commissioning errors. In Fig. 6, the ‘Ability for TPS to detect’ column addresses the probability of an error occurring. The ‘Clinical Severity’ column addresses the clinical relevance of each error, and the IROC and TG‐119 columns address the probability of the errors being detected after the fact with the current commissioning guidelines and credentialing procedures in place. The 3D Dosimetry columns then assess the possible sensitivity of the current procedures if 3D dosimetric indices were used rather than planar and point dose measurements.

Here we have investigated the ability of the TG‐119 IMRT commissioning procedures and IROC credentialing to detect various commissioning errors. It should be noted that some of these errors would likely be detected via other quality assurance tests; for example, an accurate 2nd MU calculation would likely detect a problem with the depth‐dose profiles, and inaccurate DLG might be detected during pretreatment IMRT QA. Such redundant checks will serve to minimize the probability of an undetected error.

## V. CONCLUSIONS

The AAA commissioning process within the Eclipse TPS is surprisingly robust to user error. The most severe of errors implemented was the input of TMR curves instead of PDDs. While this error had severe clinical effects, it was easily detected with IROC credentialing and TG 119 commissioning. No commissioning errors were found to have both a low detection probability and high clinical severity. When errors do occur, the IROC credentialing and TG 119 commissioning criteria are generally effective at detecting them; however, OAR TLDs are the most sensitive despite the IROC currently excluding them from analysis. IROC film analysis was ineffective in detecting the commissioning errors that we implemented. It is also critically necessary to do absolute dose plane comparison rather than relative comparison, as relative gamma analysis was unable to detect even the most dramatic of commissioning errors.
